# Factors affecting mortality in geriatric patients hospitalized with COVID-19

**DOI:** 10.3906/sag-2008-91

**Published:** 2021-04-30

**Authors:** Rabia BAĞ SOYTAŞ, Damla ÜNAL, Pınar ARMAN, Veysel SUZAN, Tuğçe EMİROĞLU GEDİK, Günay CAN, Bora KORKMAZER, Rıdvan KARAALİ, Şermin BÖREKÇİ, Mert Ahmet KUŞKUCU, Hakan YAVUZER, Deniz SUNA ERDİNÇLER, Alper DÖVENTAŞ

**Affiliations:** 1 Division of Geriatrics, Department of Internal Medicine, Cerrahpaşa Faculty of Medicine, İstanbul University-Cerrahpaşa, İstanbul Turkey; 2 Department of Public Health, Cerrahpaşa Faculty of Medicine, İstanbul University-Cerrahpaşa, İstanbul Turkey; 3 Department of Radiology, Cerrahpaşa Faculty of Medicine, İstanbul University-Cerrahpaşa, İstanbul Turkey; 4 Department of Infectious Diseases, Cerrahpaşa Faculty of Medicine, İstanbul University-Cerrahpaşa, İstanbul Turkey; 5 Department of Pulmonary Diseases, Cerrahpaşa Faculty of Medicine, İstanbul University-Cerrahpaşa, İstanbul Turkey; 6 Department of Medical Microbiology, Cerrahpaşa Faculty of Medicine, İstanbul University-Cerrahpaşa, İstanbul Turkey

**Keywords:** COVID-19, geriatrics, hospitalized patients, mortality, risk factors

## Abstract

**Background/aim:**

We aimed to investigate the factors affecting the mortality of patients aged 65 years or older who were hospitalized with the diagnosis of new coronavirus pneumonia (COVID-19).

**Materials and methods:**

This is a retrospective study of patients 65 years old or older with COVID-19 who were hospitalized in İstanbul University-Cerrahpaşa, Cerrahpaşa Medical Faculty Hospital, between March 11 and May 28, 2020. Demographic, clinical, treatment, and laboratory data were extracted from electronic medical records. We used univariate and multivariate logistic regression methods to explore the risk factors for in-hospital death.

**Results:**

A total of 218 patients (112 men, 106 women) were included, of whom 166 were discharged and 52 died in hospital. With univariate analysis, various clinical features and laboratory variables were found to be significantly different (i.e. P < 0.05). In multivariate logistic regression analysis the following were independently associated with mortality: present malignancy [odds ratio (OR) = 4.817, 95% confidence interval (CI) = 1.107–20.958, P: 0.036]; dyspnea (OR = 4.652, 95% CI = 1.473–14.688, P: 0.009); neutrophil/lymphocyte ratio (NLR; OR = 1.097, 95% CI = 1.012–1.188, P: 0.025); the highest values of C-reactive protein (CRP; OR = 1.006, 95% CI = 1.000–1.012, P: 0.049), lactate dehydrogenase (LDH; OR = 1.002, 95% CI = 1.001–1.004, P: 0.003), and creatinine levels (OR = 1.497, 95% CI = 1.126–1.990, P: 0.006); oxygen saturation (SpO2) values on admission (OR = 0.897, 95% CI = 0.811–0.993, P: 0.036); and azithromycin use (OR = 0.239, 95% CI = 0.065–0.874, P: 0.031).

**Conclusion:**

The presence of malignancy; symptoms of dyspnea; high NLR; highest CRP, LDH, and creatinine levels; and low SpO2 on admission predicted mortality. On the other hand, azithromycin use was found to be protective against mortality. Knowing the causes predicting mortality will be important to treat future cases more successfully.

## 1. Introduction

In December 2019, the pneumonia epidemic that was centered in the city of Wuhan, China, was defined as coronavirus disease 2019 (COVID-19) due to the newly defined severe acute respiratory syndrome coronavirus 2 (SARS-CoV-2) agent. This virus is thought to have been transmitted from a zoonotic infection, such as SARS-CoV or Middle East respiratory syndrome coronavirus (MERS-CoV) that is, transmitted from animals to humans. Coronaviruses are single chain, positive polarity, enveloped RNA viruses. Because of their positive polarity, they do not contain the RNA-dependent RNA polymerase enzyme; however, they encode this enzyme in their genome. They have rod-like extensions on their surfaces [1]. The disease spectrum caused by a coronavirus in humans can range from simple colds to severe acute respiratory syndrome. It may cause clinical presentations in humans and animals with various degrees of respiratory, enteric, hepatic, nephrotic, and neurological involvements [2]. The World Health Organization (WHO) accepted COVID- 19 as a global pandemic and declared an emergency on March 11, 2020. WHO (2021). WHO Timeline – COVID-19 [online]. Website https://www.who.int/news-room/detail/08-04-2020-who-timeline---covid-19 [accessed 11 March 2020]. That same day, the first case was reported by Republic of Turkey Ministry of Health.Republic of Turkey Ministry of Health (2021). COVID-19 Information Page [online]. Website https://covid19bilgi.saglik.gov.tr/depo/rehberler/COVID-19_Rehberi.pdf [accessed 11 March 2020]. Although several predictions have been made about the end of the pandemic’s progression and the occurrence of the second wave, much remains uncertain [3].

As is known, patients 65 years of age or over can be very frail due to their comorbidities and age-related physiological changes [4]. As a matter of fact, the data obtained from scientific studies have revealed that older adults are among the groups most at risk for the disease [5]. It causes higher mortality rates, especially in older people with other health problems, such as cardiovascular disease, chronic respiratory system disease, diabetes, or cancer [6]. Compared to people in a younger age group, the symptoms and course of the disease in older adults may also differ. Imperial College London’s COVID- 19 Response Team reports that those who show symptoms in their 70s are more than 20 times more likely to be hospitalized than those in their 20s [7]. In this study, we aimed to investigate the factors affecting the mortality and survival of older patients hospitalized with the diagnosis of COVID-19 in COVID inpatient clinics or the intensive care unit. Furthermore, we aimed to ensure early precautions will be taken in a second wave.

## 2. Methods

### 2.1. Participants

The participants were identified from inpatients of the İstanbul University-Cerrahpaşa, Cerrahpaşa Medical Faculty Hospital, by searching the hospital’s electronic medical records between March 11 and May 28, 2020. Beginning March 11, the facility was changed to a pandemic hospital. Patients aged 65 years or older who were diagnosed with COVID- 19 and discharged or died in hospital by May 28, 2020, were enrolled in this study. The diagnoses of all included patients were confirmed using the Diagnosis and Treatment Guideline for COVID-19 published by Republic of Turkey Ministry of Health.2 

### 2.2. Data collection

Demographic data, application complaints, comorbidity, medication use, and smoking history were extracted from electronic medical records and retrospectively reviewed and analyzed. CURB-65 scores were calculated on admission. The abbreviation stands for the following, with one point assigned for a “yes”: confusion, Urea > 41 mg/dL, respiratory rate > 30 breaths/min, blood pressure < 90/60 mm/Hg, and age ≥ 65. Complete blood count, neutrophil/lymphocyte ratio (NLR), C-reactive protein (CRP), procalcitonin, ferritin, lactate dehydrogenase (LDH), urea, creatinine, liver function tests, albumin, international normalized ratio (INR), fibrinogen, and D-dimer levels were recorded on admission, as were the highest values of CRP, creatinine, D-dimer, LDH, and ferritin levels. The number of hospitalized days at intensive care units and inpatient clinics, number of days intubated, electrocardiogram (ECG) changes, presence of delirium, presence of pressure ulcers during hospitalization, oxygen treatment types, and oxygen saturation (SpO2) values on admission were noted from the clinical course of the patient files. Also recorded were COVID-19 polymerase chain reaction (PCR) test results, thorax computed tomography (CT) findings, used medicine for the treatment of COVID-19, and adverse effects and complications. Thorax CT findings were staged by a radiology specialist in accordance with the reporting recommendations of the Radiological Society of North America Expert Consensus. According to the findings in the images, they were classified as typical, indeterminate, atypical, or negative in appearance [8]. Patients with a typical, indeterminate, or atypical appearance were accepted as thorax CT positive and received COVID treatment. The mortality and discharge rates were examined. Patients who had no fever for the last three days, whose clinical findings and laboratory values decreased, and who had been hospitalized for at least seven days were discharged.

### 2.3. Statistical analysis

Data were expressed as medians (interquartile range) for continuous variables and counts and frequencies [number (percentage)] for categorical variables. A comparison of categorical variables between the discharged and deceased groups was performed using Pearson’s χ2 test with continuity correction or Fisher’s exact test, where appropriate. The Mann–Whitney U test was used to compare differences in continuous variables between the two groups because they were all nonnormally distributed. Multivariable logistic regression modeling was used to explore independent risk factors for death. Regression analysis was performed for parameters associated with mortality within their own groups (comorbidities, symptoms, etc.). Multivariate regression analysis was also performed for the parameters that were significant in these analyses. Although some parameters, which were not associated with mortality in our study, related to mortality in the literature (age, hypertension) were also included in the multivariate regression analysis. Continuous variables were treated as continuous measures in the model. Statistical analyses were performed using SPSS version 21.0 (IBM Corporation, Armonk, NY, USA). Differences were considered to be statistically significant when two-sided P values were less than 0.05.

## 3. Results

The first cases in Turkey were reported on March 11. By May 28, the total number of cases had reached 160,979, and the total number of deaths reported was 4461.Republic of Turkey Ministry of Health (2021). COVID-19 Information Page [online]. Website https://covid19bilgi.saglik.gov.tr/depo/rehberler/COVID-19_Rehberi.pdf [accessed 28 March 2020]. Between the same dates, 1808 cases and 86 deaths related to COVID-19 were reported at İstanbul University-Cerrahpaşa, Cerrahpaşa Medical Faculty Hospital, a pandemic hospital. Of the patients, 908 were hospitalized in the COVID inpatient clinics or intensive care unit, and 251 of the inpatients were over 65 years of age. Thirty-three of these 251 geriatric patients were excluded from the study because COVID PCR or thorax CT tests could not confirm the diagnoses despite clinical findings. The other 218 patients (112 men, 106 women) were included. Sixty-eight of the patients were hospitalized in the intensive care unit and 150 in the inpatient clinics. Among them, 52 died (34 men, 18 women) specifically, 70.5% (n: 48) of the patients who were hospitalized in the intensive care unit and 2.6% (n: 4) in the inpatient clinics. Mortality was 9.5% among all COVID-19 patients (n: 86) in the inpatient clinics or intensive care unit, regardless of age, while in the geriatric group, this rate increased to 23.9%. The patients were divided into three age groups ranging from 65–74 (n: 107), 75–84 (n: 86), and 85 or more (n: 25) years. Furthermore, this study investigated sex differences by placing participants into two groups: female and male. However, no significant difference was found between the sex and age groups in terms of mortality. 

As shown in Table 1, the most common comorbidities were hypertension (66.0%), type 2 diabetes mellitus (31.7%), coronary artery disease (CAD; 31.2%), chronic obstructive pulmonary disease (COPD; 19.3%), congestive heart failure (CHF; 17.0%), and malignancy (14.7%). Of the patients, 18.8% were using an angiotensin-converting enzyme inhibitor (ACEI), and 25.7% were using an angiotensin receptor blocker (ARB). Polypharmacy was present in 49.5% of the patients, and no significant difference was found between age or sex groups. Also, 24.0% of patients had a fever, 39.0% had a cough, and 41.0% had shortness of breath. Other symptoms were less common (Table 1). 

**Table 1:** Demographics and clinical presentation in patients with COVID-19 pneumonia.

Characteristics	All patients	Recovery and discharge	Death	P value
n	218	166 (76.1 %)	52 (23.9 %)	
Age, Mean (25–75)	75.3 (70–81)	75 (69–81)	74.5 (70–80)	0.421
Age, groups, n (%) 65–74 75–84 ≥85	107 (49.1 %) 86 (39.4 %) 25 (11.5 %)	81 (48.8 %) 69 (41.6 %) 16 (9.6 %)	26 (50 %) 17 (32.7 %) 9 (17.3 %)	0.243
Sex Male n (%) Female n (%)	112 (51.4 %) 106 (48.6 %)	78 (47 %) 88 (53 %)	34 (65.4 %) 18 (34.6 %)	0.021
Comorbidity n (%)				
Hypertension	145 (66.5 %)	113 (68.1 %)	32 (61.5 %)	0.403
Diabetes	69 (31.7 %)	57 (34.3 %)	12 (23.1 %)	0.128
CAD	68 (31.2 %)	49 (29.5 %)	19 (36.5 %)	0.340
CHF	37 (17 %)	23 (13.9 %)	14 (26.9 %)	0.035
Malignancy	32 (14.7 %)	14 (8.4 %)	18 (34.6 %)	<0.001
COPD	42 (19.3 %)	28 (16.9 %)	14 (26.9 %)	0.109
Chronic renal disease	28 (12.8 %)	19 (11.4 %)	9 (17.3 %)	0.270
Dementia	24 (11 %)	14 (8.4 %)	10 (19.2 %)	0.03
Cerebrovascular disease	19 (8.7 %)	13 (7.8 %)	6 (11.5 %)	0.406
Polypharmacy	108 (49.5 %)	75 (45.2 %)	33 (63.5 %)	0.021
ACEI n (%)	41 (18.8 %)	29 (17.5 %)	12 (23.1 %)	0.367
ARB n (%)	56 (25.7 %)	48 (28.9 %)	8 (15.4 %)	0.051
Smoking	44 (20.2 %)	30 (18.1 %)	14 (26.9 %)	0.165
Presence of symptoms, n (%)	207 (95 %)	156 (94 %)	51 (98.1 %)	0.238
Fever	53 (24.3 %)	41 (24.7 %)	12 (23.1 %)	0.812
Cough	85 (39 %)	75 (45.2 %)	10 (19.2 %)	0.001
Dsypnea	90 (41.3 %)	55 (33.1 %)	35 (67.3 %)	<0.001
Fatigue	28 (12.8 %)	23 (13.9 %)	5 (9.6 %)	0.425
Known contact history	17 (7.8 %)	15 (9 %)	2 (3.8 %)	0.373
Nausea/vomiting	10 (4.6 %)	8 (4.8 %)	2 (3.8 %)	0.770
Loss of consciousness	10 (4.6 %)	6 (3.6 %)	4 (7.7 %)	0.255
Sore throat	9 (4.1 %)	9 (5.4 %)	0	0.119
Myalgia	8 (3.7 %)	8 (4.8 %)	0	0.107
Diarrhea	6 (2.8 %)	4 (2.4 %)	2 (3.8 %)	0.581
Chills	6 (2.8 %)	5 (3 %)	1 (1.9 %)	0.675
Headache	1 (0.5 %)	1 (0.6 %)	0	0.575

The patients’ laboratory values on admission and the highest values are shown in Table 2. The COVID PCR tests were positive in 46.7% of the patients, and COVID findings were positive in thorax CTs in 91.3% of the patients. For 83 patients (38.0%), both their COVID PCR and thorax CT tests were positive. In 19 patients (8.7%), COVID PCR was positive while thorax CT was negative, and in 116 patients (53.2%), COVID PCR was negative while thorax CT was positive. The blood pressure of 5.0% of the patients was greater than 90/60 mmHg on admission. 

**Table 2:** Laboratory findings in patients with COVID-19 pneumonia.

Laboratory results, mean (25–75)	All patients	Recovery and discharge	Death	P value
Hemoglobin, g/dL	11.9 (10.6–13.1)	12.2 (10.6–13.3)	11.3 (10.3–12.2)	0.073
WBC, 10^3/μL	7.45 (5.3–10.7)	6.9 (5–8.8)	10.5 (7.4–13.5)	0.001
Platelets, 10^3/μL	211000 (160550–263550)	217000 (160900–263100)	196800 (148725–277500)	0.191
Lymphocyte, 10^3/μL	1.2 (0.8–1.7)	1.3 (0.9–1.9)	0.9 (0.5–1.3)	0.002
NLR, 10^3/μL	4.15 (2.27–8.78)	3.3 (1.8–5.2)	10.7 (5–18.2)	<0.001
Ferritin, ng/mL	279.9 (102–685.7)	231 (91.7–582)	532 (282–1459)	0.001
Highest ferritin, ng/mL	516.6 (189.6–1264)	354.3 (165.8–822.5)	1543 (690–2000)	0.001
D-dimer, mg/L	1.45 (0.69–3.8)	1.17 (0.5–3.05)	3.4 (1.3–7.8)	0.052
Highest D-dimer, mg/L	3.97 (1.48–12.07)	2.69 (1.14–7.21)	11.9 (6.4–34.3)	<0.001
Procalcitonin, ng/mL	0.13 (0.06–0.43)	0.09 (0.04–0.26)	0.55 (0.23–1.76)	0.260
LDH, IU/L	279.5 (210–405)	249 (201–352)	419 (293–638)	<0.001
Highest LDH, IU/L	432 (304–697.7)	405 (269.7–559.2)	726 (488–1185)	<0.001
CRP, mg/L	53 (21.7–120)	36 (147–947)	132 (53–177)	<0.001
Highest CRP, mg/L	120.1 (42.7–231.9)	82.14 (33.01–162.6)	260 (210–325)	<0.001
INR	1.1 (1.03–1.2)	1.1 (1.01–1.2)	1.2 (1.1–1.4)	0.013
Urea, mg/L	90 (86–94)	9 (7–14.2)	12 (5.2–18)	<0.001
Creatinin, mg/L	1.02 (0.8–1.4)	1 (0.78–1.26)	1.24 (0.8–1.97)	0.04
Highest creatinin, mg/L	1.33 (0.95–2.02)	1.19 (0.91–1.61)	1.24 (0.8–1.97)	<0.001
AST, IU/L	29.5 (19–45)	25.8 (18–38)	45 (30–57)	0.004
ALT, IU/L	17 (12–32)	16 (12–28)	23 (12.5–56.5)	0.038
Fibrinogen, mg/dL	456 (357–573)	451 (346–557)	499 (395–582)	0.088
Albumin, gr/dL	3.64 (3.2–4)	3.75 (3.3–4.05)	3.18 (2.84–3.79)	<0.001

The oxygen treatment types applied to patients during hospitalization are shown in Table 3. During hospitalization, ECG changes were observed in 18.3%, delirium in 12.4%, and pressure ulcers in 3.2% of the patients. For COVID-19 treatment, 100% of patients used hydroxychloroquine, 78.0% azithromycin, 70.6% oseltamivir, 58.3% favipiravir, 7.3% lopinavir/ritonavir, 12.4% tocilizumab, 7.8% corticosteroid, and 78.4% anticoagulants. Adverse drug reactions occurred in 8.7% of the patients, and the majority of these adverse effects were prolonged QT on ECG (6.9%).

**Table 3:** Clinical findings and treatment modalities in patients with COVID-19 pneumonia.

Clinical findings and treatment modalities	All patients		Recovery and discharge	Death	P value
Hospitalized days numbers in intensive care unit	10.09 (4–14)		8 (3.25–11.7)	10 (4–16.75)	<0.001
Intubated days numbers	9.05 (2.75–13.25)		5.5 (1.75–8)	8.5 (3–14)	<0.001
Total hospitalized days numbers	10 (7–15.25)		9 (7–14.25)	12 (5.25–18)	0.326
SpO2 % on admission	94 (89–97)		95 (91–97)	88 (77–94)	<0.001
Blood pressure on admission, n (%) ≤ 90/60 <90/60	14 (6.4 %) 204 (93.6)		11 (6.6 %) 155 (93.4 %)	3 (5.7 %) 49 (94.3 %)	0.493
CURB65, n (%) 1–234–5	156 (71.6%)41 (18.8 %)21 (9.6 %)		135 (81.3 %)25 (15.1 %)6 (3.6 %)	21 (40.4 %)16 (30.8 %)15 (28.8 %)	<0.001
COVID PCR positive, n (%)	102 (46.7 %)		75 (45 %)	27 (51.9 %)	0.561
Thorax CT positive, n (%)	199 (91.3 %)		151 (91 %)	48 (92.3 %)	0.06
Thorax CT Classification, n (%) 0 (negative) 1 (atypical) 2 (indeterminate) 3 (typical)	19 (8.7 %) 24 (11 %) 40 (18.3 %) 135 (61.9 %)		15 (9 %) 14 (8.4 %) 30 (18.1 %) 107 (64.5 %)	4 (7.6 %) 10 (19.2 %) 10 (19.2 %) 28 (53.8 %)	0.094
Oxygen treatment, n (%)					
Nasal		126 (57.8 %)	98 (59 %)	28 (46.2 %)	0.508
Mask with a reservoir		26 (11.9 %)	7 (4.2 %)	19 (36.5 %)	<0.001
High Flow		15 (6.9 %)	9 (5.4 %)	6 (11.5 %)	0.204
NIMV		24 (11 %)	8 (4.8 %)	16 (30.8 %)	<0.001
IMV		57 (26.1 %)	10 (6 %)	47 (90.4 %9	<0.001
Delirium		27 (12.4 %)	19 (11.5 %)	8 (15.7 %)	0.431
Pressure ulcer		7 (3.2 %)	5 (3 %)	2 (3.8 %)	0.766
ECG change		40 (18.3 %)	28 (16.9 %)	12 (23.1 %)	0.313
COVID treatment, n (%)					
Hydroxychloroquine		215 (98.6 %)	166 (100 %)	52 (100 %)	0.425
Azithromycin		170 (78%)	136 (82.4 %)	34 (65.4 %)	0.009
Oseltamivir		154 (70.6 %)	117 (71.3 %)	37 (71.2 %)	0.979
Favipiravir		127 (58.3 %)	80 (48.5 %)	47 (90.4 %)	<0.001
Lopinavir/ritonavir		16 (7.3 %)	13 (7.8 %)	3 (5.8 %)	0.619
Tocilizumab		27 (12.4 %)	22 (13.3 %)	5 (9.6 %)	0.479
Anticoagulants		171 (78.4 %)	132 (79.5 %)	39 (75 %)	0.635
Corticosteroids		17 (7.8 %)	10 (6 %)	7 (13.5 %)	0.081

Thus far, this results section has profiled this study’s 218 geriatric patients. Now, the focus shifts to the relationships between mortality and the patients’ characteristics. The mortality rate of men was higher than that of women (P: 0.021). However, no significant relationship was found between age groups and death. In terms of comorbidities, although our study found no such relationship, some epidemiological studies have shown that hypertension is associated with increased mortality and morbidity in COVID-19 patients. On the other hand, our study did find CHF, malignancy, and dementia to be more common in patients who died (respectively, P: 0.035, < 0.001, and 0.03). Polypharmacy was found in 49.5% of the patients and was significantly higher in the patients who died (P: 0.021).

There was no relationship between the excess number of symptoms and death. When the symptoms were evaluated one by one, a significant relationship was found between cough and death and between dyspnea and death (respectively, P: 0.001 and < 0.001). 

This study also investigated possible relationships between mortality and laboratory values. If the patient’s levels of lymphocytes were lower and the white blood cell count and NLR were higher on admission, these changes in values were significantly related to mortality (respectively, P: 0.001, 0.002, and < 0.001). CRP, ferritin, and LDH on admission were all associated with mortality (respectively, P: < 0.001, 0.001, and < 0.001), as were their highest values (respectively, P: < 0.001, 0.001, and < 0.001). The highest value of D-dimer was also associated with mortality (P: < 0.001). In patients who died, INR, aspartate aminotransferase (AST), and alanine aminotransferase (ALT) values on admission were significantly higher, and albumin values were lower. Urea and creatinine levels on admission and the highest creatinine values were higher in the patients who died (respectively, P: < 0.001, 0.04, and < 0.001).

CURB-65 is a severity score containing five variables used to determine the severity of community-acquired pneumonia [9]. In this study, mortality was significantly higher in patients with a CURB-65 score of four to five than in other groups (P: < 0.001). It was observed that patients with lower baseline SpO2 values (P: < 0.001) and whose CURB-65 scores were four to five died more (P < 0.001). COVID PCR and thorax CT positivity were not associated with mortality.

As predicted, it was found that patients died more if they had a high number of days of hospitalization and intubation in the intensive care unit and used noninvasive and invasive mechanical ventilators. However, no relationship was found between the total number of hospitalization days and mortality. The use of a mask with a reservoir was associated with mortality (P: < 0.001).

It was found that patients who used azithromycin, one of the drugs used for COVID- 19 treatment, died less (P: 0.009). In contrast, the use of favipiravir was found to be higher in the patients who died (P: < 0.001).

In multivariate logistic regression analysis to detect the factors predicting mortality among older patients with COVID-19, malignancy; dyspnea; NLR; the highest values of CRP, creatinine, and LDH; azithromycin use; and SpO2 values on admission were independently associated with mortality (Table 4).

**Table 4:** Logistic regression analysis of COVID-19 mortality risk factors.

	Univariate analysis		Multivariate analyses		
Variable	OR (95% CI)	P value		OR (95% CI)	P value
Age, year	1.018 (0.976–1.062)	0.410		0.991 (0.913–1.075)	0.819
Sex	0.469 (0.246–0.897)	0.022		0.614 (0.186–2.022)	0.423
Hypertension	0.750 (0.393–1.433)	0.384		0.317 (0.79–1.274)	0.106
Malignancy	5.748(2.605–12.681)	<0.001		4.817(1.107–20.958)	0.036
Dementia	2.585 (1.072–6.235)	0.035		3.252(0.673–15.711)	0.142
Dsypnea	4.155 (2.140–8.067)	<0.001		4.652(1.473–14.688)	0.009
Polypharmacy	2.107 (1.109–4.004)	0.023		1.741 (0.475–6.384)	0.403
NLR, 10^3/μL	1.192 (1.122–1.267)	<0.001		1.097 (1.012–1.188)	0.025
Highest D-dimer, mg/L	1.049 (1.026–1.072)	<0.001		1.001 (0.961–1.042)	0.968
Highest CRP, mg/L	1.014 (1.01–1.018)	<0.001		1.006 (1.000–1.012)	0.049
Highest LDH, IU/L	1.003 (1.002–1.005)	<0.001		1.002 (1.001–1.004)	0.003
Highest creatinin, mg/L	1.627 (1.283–2.063)	<0.001		1.497 (1.126–1.990)	0.006
SpO2 % on admission	0.850 (0.799–0.903)	<0.001		0.897 (0.811–0.993)	0.036
Azithromycin	0.403 (0.200–0.809)	0.011		0.239 (0.065–0.874)	0.031

## 4. Discussion

This study investigated factors affecting mortality in hospitalized older adult patients with COVID-19. Many studies have shown that mortality increases with increasing age [10]. In a study with 72,314 cases, the overall mortality rate was 2.3%, but it was 8% in patients 70–79 years old and 14.5% in patients ≥ 80 years old [11]. In another analysis by the Korean Centers for Disease Control and Prevention, the overall case fatality rate (CFR) in 11,344 patients with confirmed cases on May 28, 2020, was 2.4%; however, it was much higher in older adults (10.9% in patients 70–79 years and 26.6% in patients ≥ 80 years). Republic of Korea (2021). Coronavirus Disease-19 [online]. Website http://ncov.mohw.go.kr/bdBoardList_Real.do?brdId=1&brdGubun=11&ncvContSeq=&contSeq=&board_id=&gubun [accessed 28 May 2020]. In a report published by the Italian Institute of Higher Health, the total CFR on March 26, 2020, was four times higher than in Korea or China (9.2%); however, the increase in mortality with age was similar to that in Korea and China. CFR was less than 1% in the 50–69 age group, 16.9% in the 70–79 age group, and 24.4% in the 80 and older age group. Istituto Superiore di Sanità (2021). Epidemia COVID-19 [online]. Website https://www.epicentro.iss.it/coronavirus/bollettino/Bollettino-sorveglianza-integrata-COVID-19_2-aprile-2020.pdf [accessed 02 April 2020]. In our study, no significant difference was found between mortality and age groups. This may be due to the fact that the number of patients in the 85 years and older age group is lower than the other groups. Also, another reason may be that comorbidities are independent of age.

The COVID-19 mortality rate was higher in male patients than female patients in this study (P: 0021). This finding contradicts another study concerning geriatric patients and the factors affecting mortality, which found no significant relationship between sex and death [12]. Furthermore, our study’s multivariate regression analysis did not point to sex as a factor that predicts mortality. However, in a large-scale study involving 1663 patients, mortality was higher in men than in women, as in our study [13]. In addition, previous studies have reported that men are more affected by SARS and MERS infections. One of the reasons for this situation may be that women have different immune responses due to sex or pay more attention to leading a healthy life. Another reason may be that malignancy was detected more frequently in males in our patient group (P: 0.033), and malignancy was associated with mortality.

Among the comorbidities, malignancy was significantly associated with mortality (OR = 4.817, 95% CI = 1.107–20.958, P: 0.036), which was consistent with other studies’ findings. A study comparing COVID-19 patients with and without cancer history found that malignancy was an independent risk factor for mortality, similar to our study [14]. This result may be due to the different immune and inflammatory responses of patients with a history of cancer.

The angiotensin-converting enzyme 2 (ACE2) has been shown to be a coreceptor for viral entry of SARS-CoV-2, suggesting that it has a role in the pathogenesis of COVID-19 [15]. Therefore, ACEI and ARB use are thought to increase COVID-19 mortality and morbidity. However, some studies have shown that ACEIs prescribed at the clinic do not inhibit ACE2, which acts as a carboxypeptidase [16]. Similarly, in our study, no relationship was found between ACEI and ARB use and mortality.

In a study comparing patients infected with COVID-19 with and without dementia, dementia was significantly associated with mortality [17]. Although regression analysis did not predict mortality in our study, it was observed that dementia was significantly higher in patients who died. Since it is difficult to identify symptoms of atypical COVID- 19 in dementia patients, the interval between the onset of symptoms and hospital admission may have been prolonged, which may have adversely affected the prognosis. 

There is no clear consensus for polypharmacy, and there are different definitions. Some of these definitions are the use of two or more drugs within 240 days, using four or more drugs, or using five or more drugs [18]. Since the use of five or more drugs is a more accepted definition, we defined polypharmacy as the use of five or more drugs in our study. There is no research in the literature investigating the effect of polypharmacy on COVID-19 prognosis. In our study, while polypharmacy was found to be significantly high in patients who died, it was not one of the parameters predicting mortality in multivariate regression analysis.

Symptoms were present in 95.0% of the patients, but there was no relationship between the presence of symptoms and mortality. Since almost all the patients had symptoms, we can say that there was no significant relationship between mortality and whether there were symptoms. Although fever is the most common symptom in the general population (98.0%) [19], the most common symptom in our study was dyspnea (41.0%). Due to decreased immune system response in older adults, fever response to infections is also generally reduced. In our study, 24.0% of patients had a fever, which is a much lower percentage than the overall COVID-19 patients. Among the symptoms, a significant relationship was only found between dyspnea and mortality (OR = 4.652, 95% CI = 1.473–14.688, P: 0.009). Dyspnea in those who died was twice as frequent as in those who were discharged. This result may indicate that a COVID-19 patient presenting with dyspnea will have a worse course of infection than another patient without dyspnea. 

There are studies in the literature about the laboratory predictors of death from COVID- 19. In our study’s multivariate regression analysis, it was determined that NLR and the highest values of CRP, creatinine, LDH levels may predict mortality. NLR is a marker of systemic inflammation predicting prognosis in various pathological conditions [20]. Recently, in community-acquired pneumonia, NLR has been found to have more prognostic power than known infection markers, such as CRP, white blood cell count, and neutrophil count [21]. There are also studies showing that the lymphocyte count affects the prognosis of COVID-19 disease [22]. Research has reported that severe COVID-19 cases had a higher neutrophil count and lower lymphocyte count than nonsevere patients, so NLR tends to be higher in patients with severe infection [23]. In addition to these studies, our study found NLR to be one of the predictors of mortality in COVID-19 patients (OR = 1.097, 95% CI = 1.012–1.188, P: 0.025). NLR may be a parameter that can be easily used to predict mortality since it can be calculated with a simple hemogram test. Some studies have reported that an elevated CRP is related to COVID-19 mortality [24]. In this study, the highest CRP values were associated with mortality (OR = 1.006, 95% CI = 1.000–1.012, P: 0.049). A cytokine storm occurs during virus invasion and can interact with excessive neutrophil infiltration, leading to the formation of harmful inflammatory processes. The immune system is suppressed, and the T lymphocyte response decreases. As a result, accompanying bacterial infections occur. These conditions may be the reason why elevated CRP and NLR predict mortality.

Similar to some studies, in this study, the relationship between high D-dimer values and mortality was determined [25]. Research has shown that the risk of both arterial and venous thromboembolism increases, especially in patients with a severe course of COVID-19 [26]. This may be the reason why a high D-dimer level is found to be associated with mortality. In response, anticoagulants have been included in the COVID-19 treatment protocols. Higher LDH values are not surprising in COVID-19 patients who died (OR = 1.002, 95% CI = 1.001–1.004, P: 0.003). There is reliable evidence that increased LDH values reflect the degree of pulmonary damage in patients with acute respiratory distress syndrome. Also, these data have been reported with SARS [27]. It was reported that multiorgan failure may be seen in severe COVID-19 cases [28]. The fact that the highest creatinine values in our study were associated with mortality may actually be a result of serious disease (OR = 1.497, 95% CI = 1.126–1.990, P: 0.006).

It is already known that hypoxemia is a sign of poor prognosis in pulmonary diseases, and the indicators for hypoxemia are used to evaluate the severity of COVID-19 [19]. Therefore, it is not surprising that the lower SpO2 values on admission predicted mortality in our study (OR = 0.897, 95% CI = 0.811–0.993, P: 0.036). A mask with a reservoir is used in inpatient clinics to stave off transferring to intensive care unit those patients who worsen in the service follow-up and whose SpO2 with nasal oxygen does not increase. In our study, there was a higher mortality rate in patients using a mask with reservoir (P: < 0.001). It may result from delaying the patient’s timely arrival in intensive care, or from using the mask on patients whose clinic situations have already deteriorated.

In a study comparing patients who did not use any medication to patients who used azithromycin, hydroxychloroquine, or both azithromycin and hydroxychloroquine, using azithromycin alone or in combination with hydroxychloroquine did not affect mortality [29]. Another study found that mortality increased in those using only hydroxychloroquine compared to those using hydroxychloroquine and azithromycin [30]. Similarly, in our study, patients who used azithromycin died less, and azithromycin use had a protective effect on mortality in the multivariate logistic regression analysis (OR = 0.239, 95% CI = 0.065–0.874, P: 0.031). Azithromycin is effective against Zika and Ebola viruses in vitro [31,32]. It prevents serious respiratory infections when administered to patients with viral infections. It can be protective against mortality for both this reason and because it can prevent bacterial superinfections. Favipiravir use was found to be higher in patients who died (P: < 0.001). This may be due to the fact that favipiravir has been given to patients whose SpO2 and clinical situation have deteriorated despite the classic triple treatment (hydroxychloroquine, azithromycin, and oseltamivir).

In this study, in which the factors affecting mortality in geriatric patients with COVID-19 were investigated, appropriate and contributing data were obtained from the literature. The limitations of our study were that the number of patients in the three age groups was dissimilar, and information about the time between a patient’s onset of complaints and admission to the hospital was not available in the files. Our study needs to be supported by prospective studies, which will be done by evaluating frailty, an essential parameter in geriatric patients.

In conclusion, the COVID-19 outbreak has been declared a global pandemic. The future course of COVID-19, when the first wave will end, and whether there will be a second wave are all important questions. The answers are not yet known. However, it is a known fact that COVID-19 is more serious and fatal in the older adults. Therefore, knowing the causes predicting mortality is important so that health care providers and others can be more conscious of them in future cases. Older patients with COVID-19 should be monitored more carefully, and more care should be taken in factors affecting mortality reported in our study and other studies in the literature. 

## Contribution of authors

Concept and design: Alper Döventaş, Hakan Yavuzer, Deniz Suna Erdinçler and Rabia Bağ Soytaş. Data collection: Damla Ünal, Pınar Arman, Veysel Suzan, Tuğçe Emiroğlu, Bora Korkmazer and Rabia Bağ Soytaş. Analysis and interpretation of data: Günay Can, Hakan Yavuzer, Alper Döventaş and Rabia Bağ Soytaş. Manuscript preparation: All authors.

## Informed consent

This study was approved by the Ethical Review Committee of **İ** stanbul University Cerrahpaşa, Cerrahpaşa Faculty of Medicine. Ethical Review Committee decision date and number: 05/06/2020-68153.

Informed consent was not obtained from the patients because the study was retrospective.
